# Supplementary material for: A Two-Stage Reconstruction of Microstructures with Arbitrarily Shaped Inclusions
Source: Materials (Basel). 2020 Jun 17;13(12):2748. doi: 10.3390/ma13122748 (PMC7345931; doi:10.3390/ma13122748)
Supplement: Supplementary file 1 [file materials-13-02748-s001.pdf]

materials

MDPI

Supplementary Material

A Two-Stage Reconstruction of Microstructures with Arbitrarily Shaped Inclusions

Ryszard Piasecki [1,*], Wiesław Olchawa [1], Daniel Frączek [2] and Agnieszka Bartecka [1]

[1] Institute of Physics, University of Opole, Oleska 48, 45-052 Opole, Poland; wolch@uni.opole.pl (W.O.); jazgara@uni.opole.pl (A.B.)
[2] Department of Materials Physics, Opole University of Technology, Katowicka 48, 45-061 Opole, Poland; dfracz@gmail.com
* Correspondence: piaser@uni.opole.pl

Received: 28 April 2020; Accepted: 13 June 2020; Published: date

Table S1. Shape libraries to Example_1.

Cluster #	Target	Seed 1	Seed 2	Seed 3
1	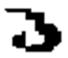	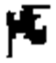	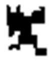	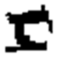
2	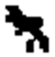	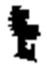	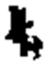	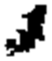
3	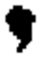	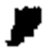	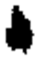	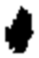
4	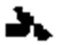	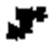	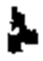	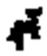
5	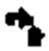	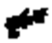	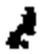	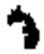

materials

Cluster #	Target	Seed 1	Seed 2	Seed 3
6	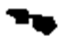	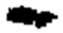	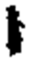	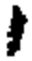
7	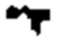	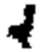	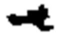	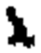
8	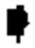	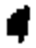	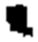	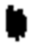
9	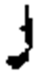	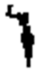	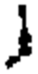	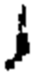
10	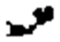	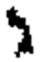	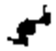	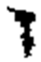
11	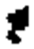	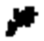	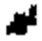	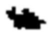
12	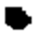	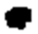	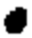	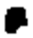

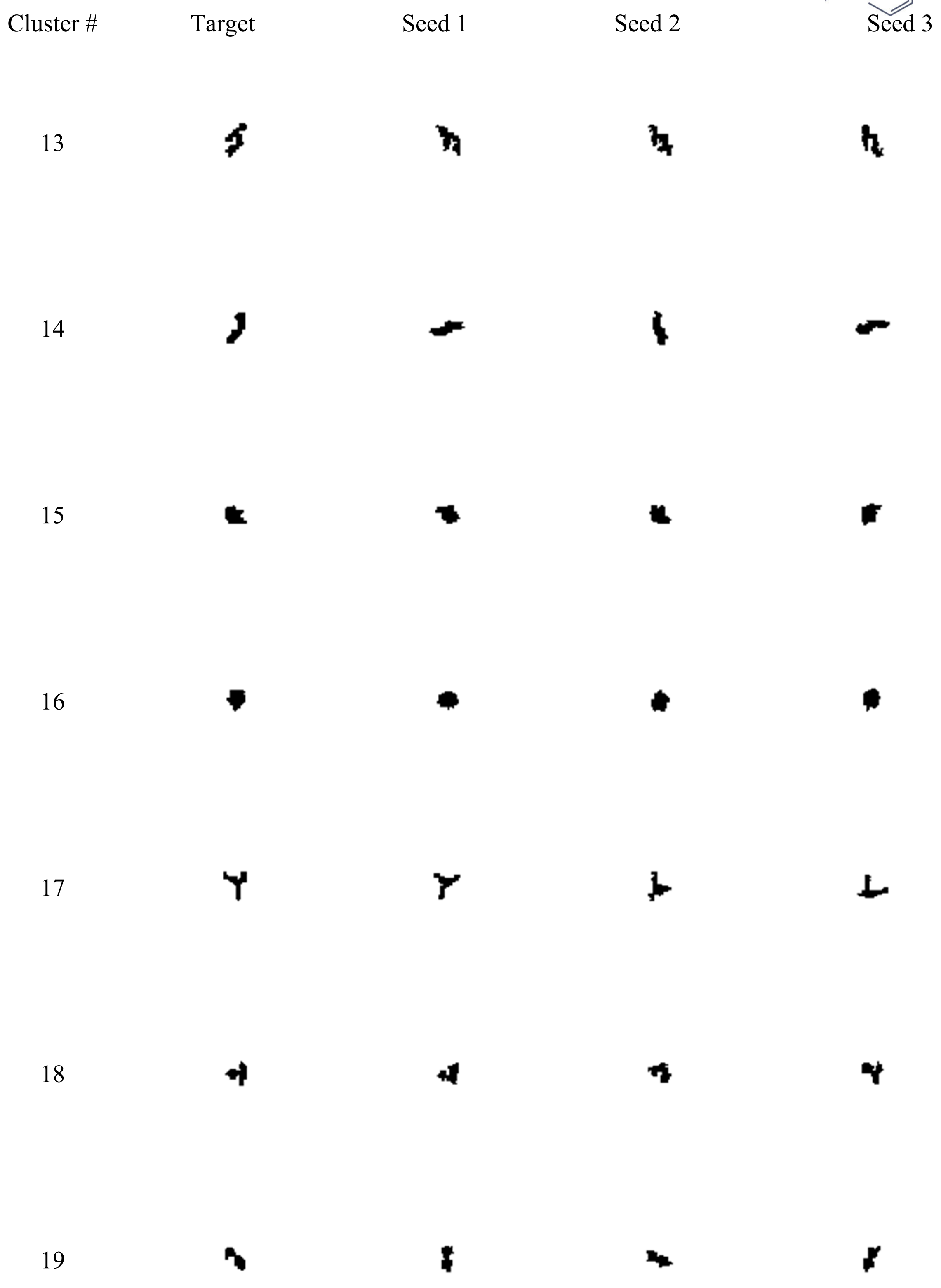
Cluster #
Target
Seed 1
Seed 2
Seed 3
13
14
15
16
17
18
19

materials
MDPI

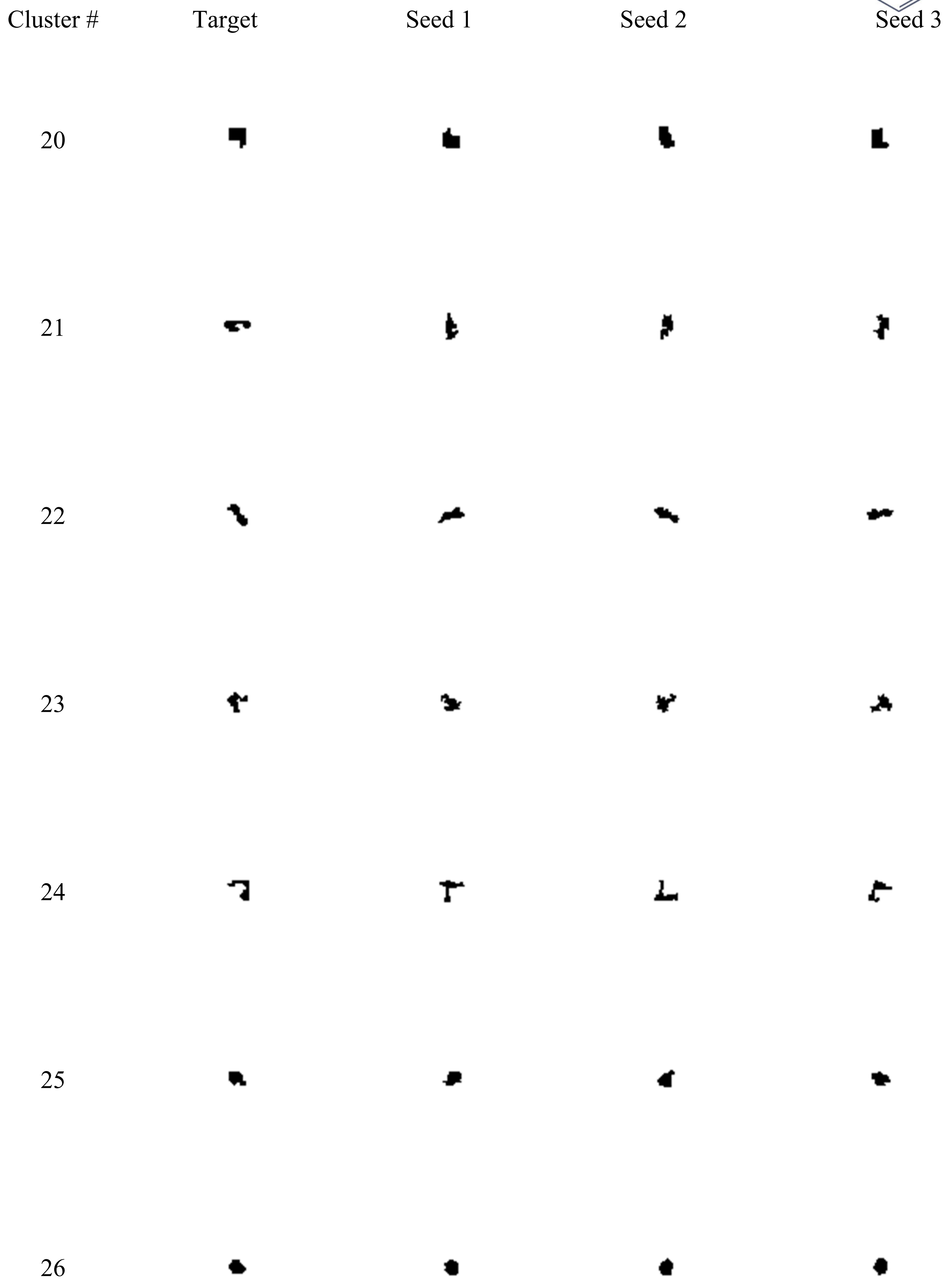

materials

Cluster #	Target	Seed 1	Seed 2	Seed 3
27	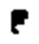	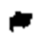	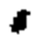	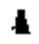
28	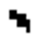	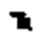	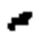	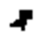
29	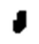	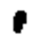	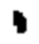	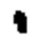
30	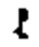	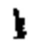	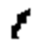	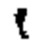
31	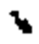	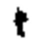	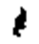	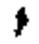
32	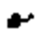	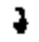	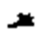	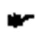
33	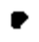	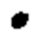	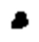	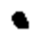

materials MDPI

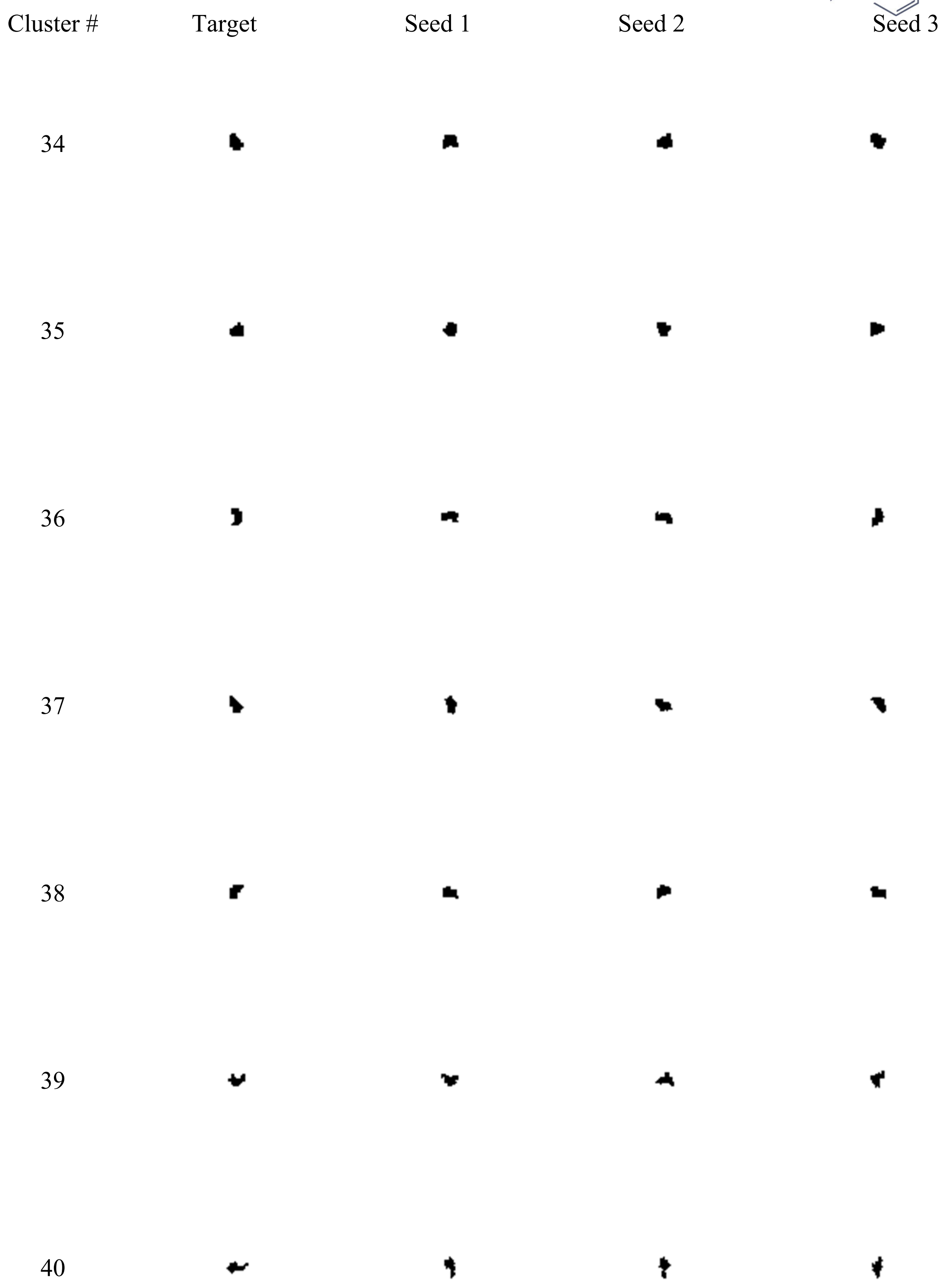

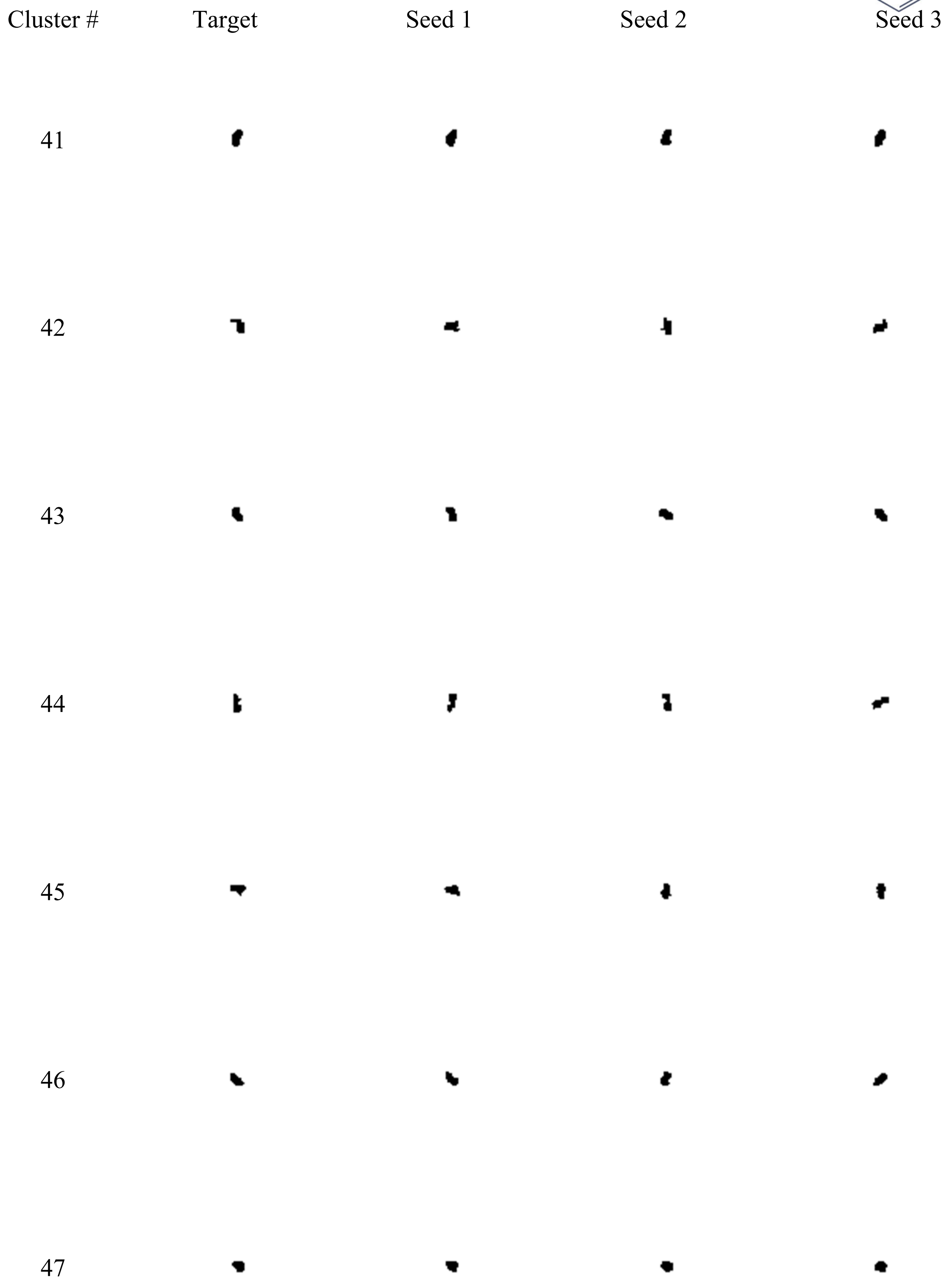
Cluster #
Target
Seed 1
Seed 2
Seed 3
41
42
43
44
45
46
47

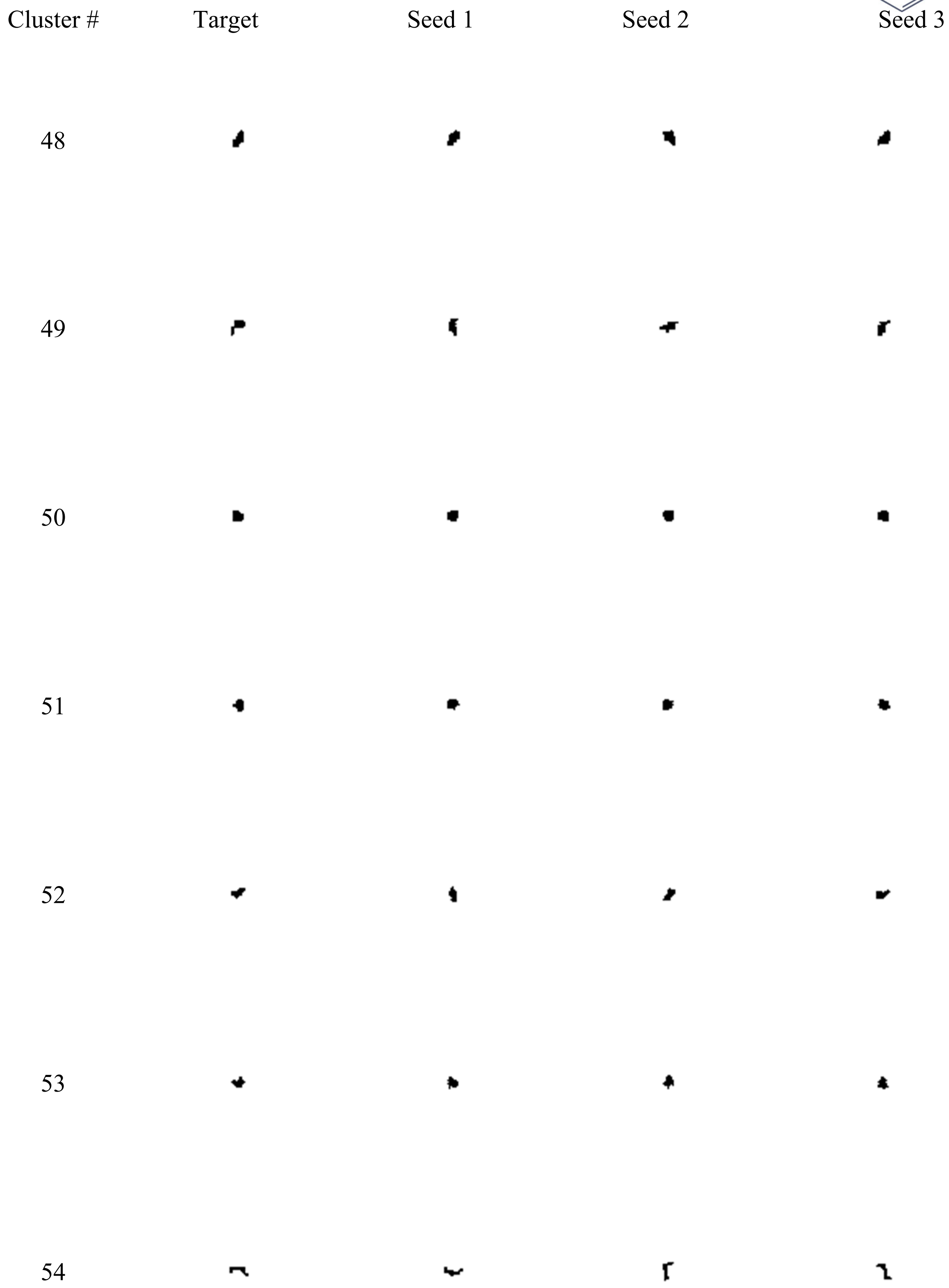
Cluster #
Target
Seed 1
Seed 2
Seed 3
48
49
50
51
52
53
54

materials

MDPI

Cluster #	Target	Seed 1	Seed 2	Seed 3
55	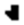	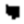	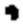	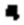
56		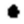		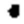
57	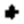	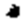	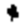	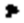
58	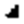	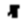		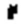
59	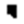	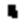		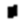
60				
61				

materials MDPI

Cluster #	Target	Seed 1	Seed 2	Seed 3
62		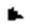	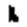	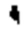
63	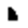	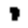		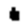
64	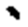	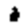	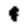	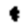
65	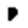			
66				
67				
68	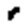			

materials

MDPI

Cluster #	Target	Seed 1	Seed 2	Seed 3
69				
70				
71				
72				
73				
74				
75	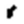			

materials

Cluster #	Target	Seed 1	Seed 2	Seed 3
76				
77				
78				
79				
80				
81				
82				

Cluster #	Target	Seed 1	Seed 2	Seed 3
83				
84				
85				
86				
87				
88				
89				

materials

Cluster #	Target	Seed 1	Seed 2	Seed 3
90				
91				
92				
93				
94				
95				
96				

Cluster #	Target	Seed 1	Seed 2	Seed 3
97				
98				
99				
100				
101				
102				
103				

materials

MDPI

Cluster #	Target	Seed 1	Seed 2	Seed 3
104				
105				
106				
107				
108				
109				
110				

materials

MDPI

Cluster #	Target	Seed 1	Seed 2	Seed 3
111				
112				
113				

materials

MDPI

Table S2. Shape libraries to Example_2.

Cluster #	Target	Seed 1	Seed 2	Seed 3
1	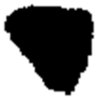	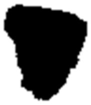	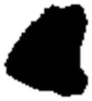	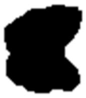
2	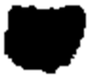	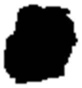	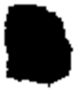	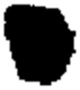
3	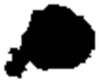	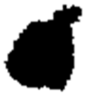	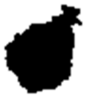	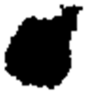
4	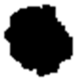	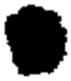	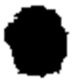	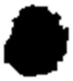
5	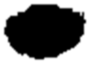	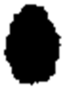	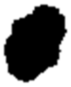	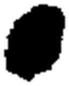

materials

MDPI

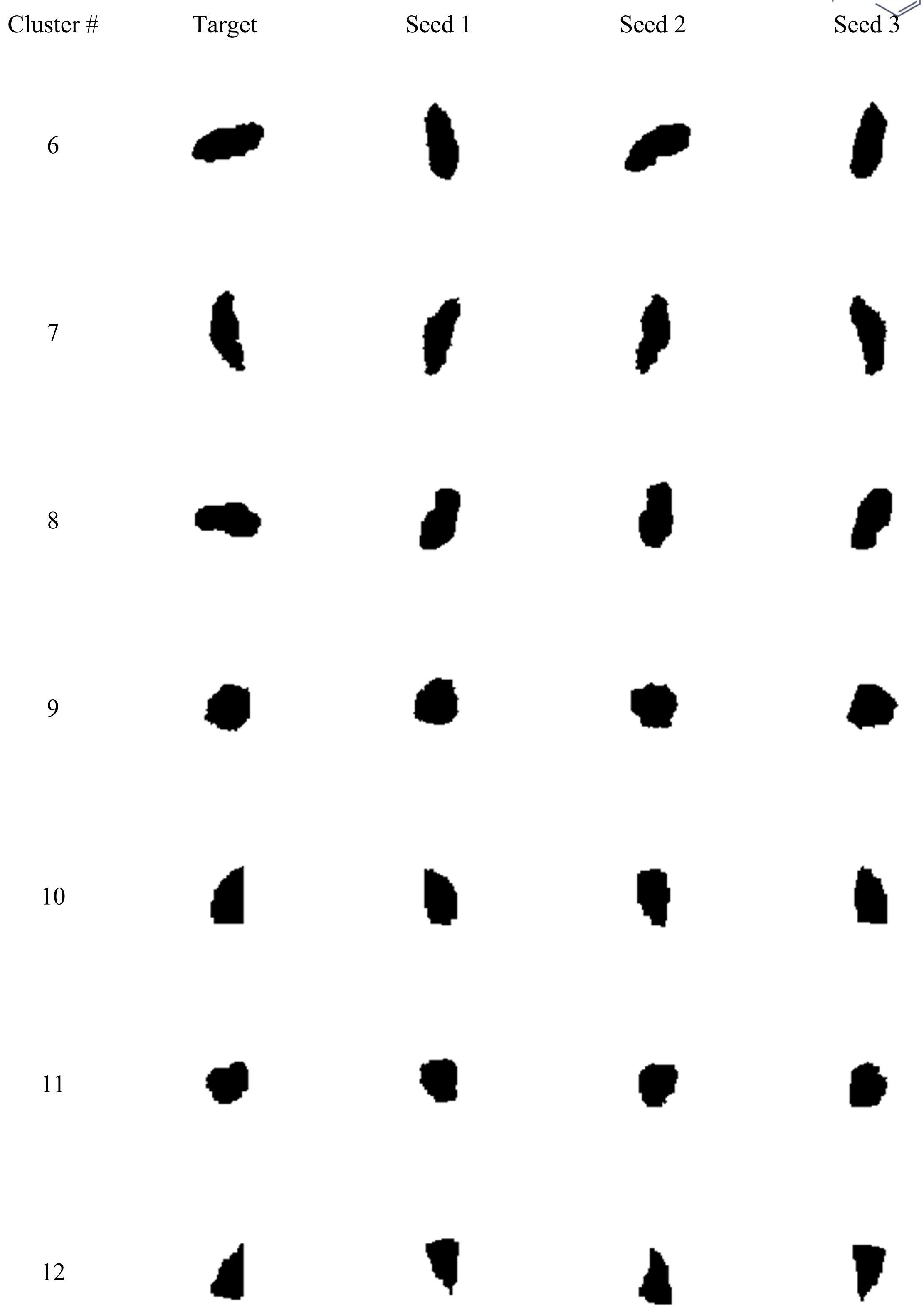

materials
MDPI

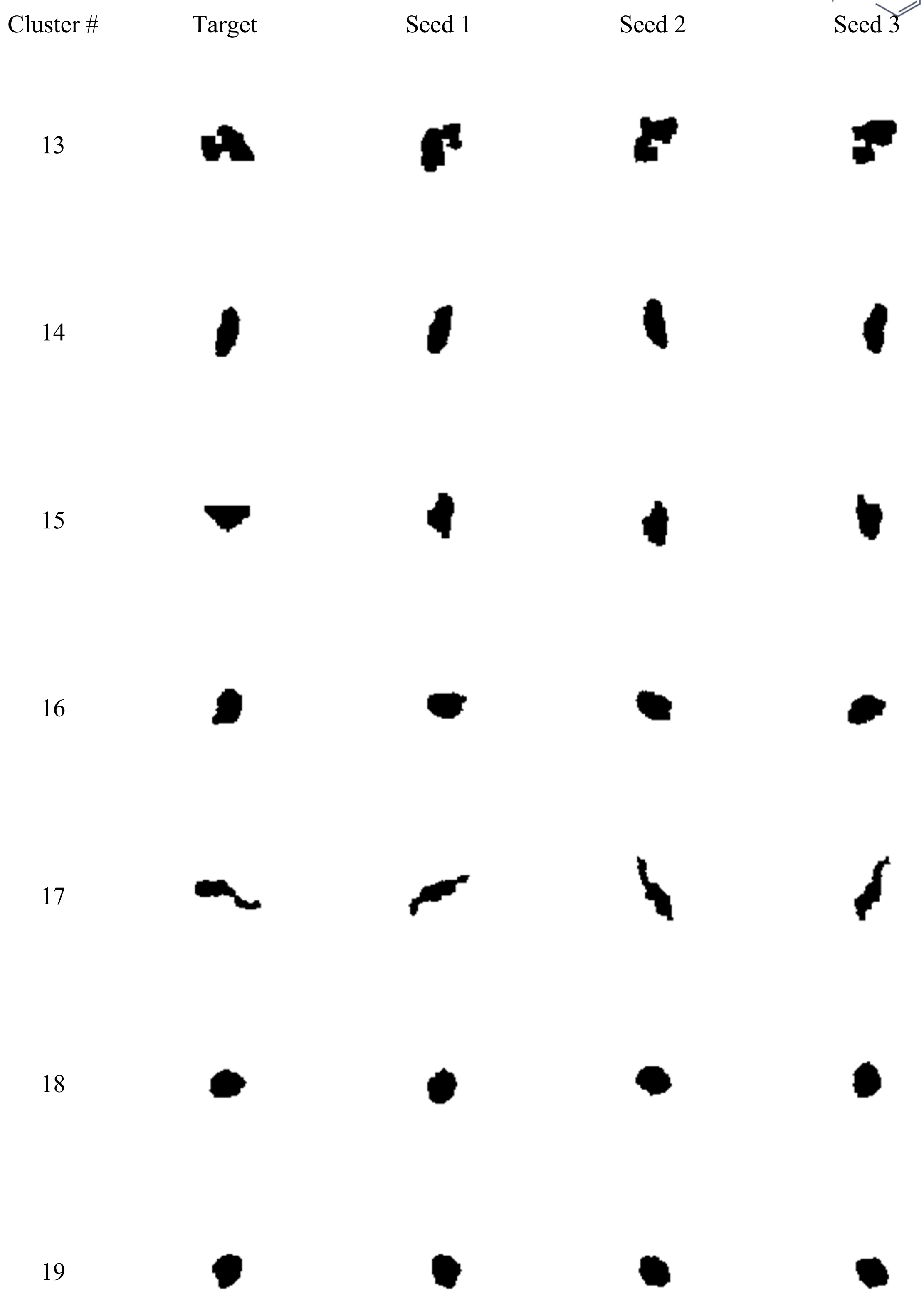

materials

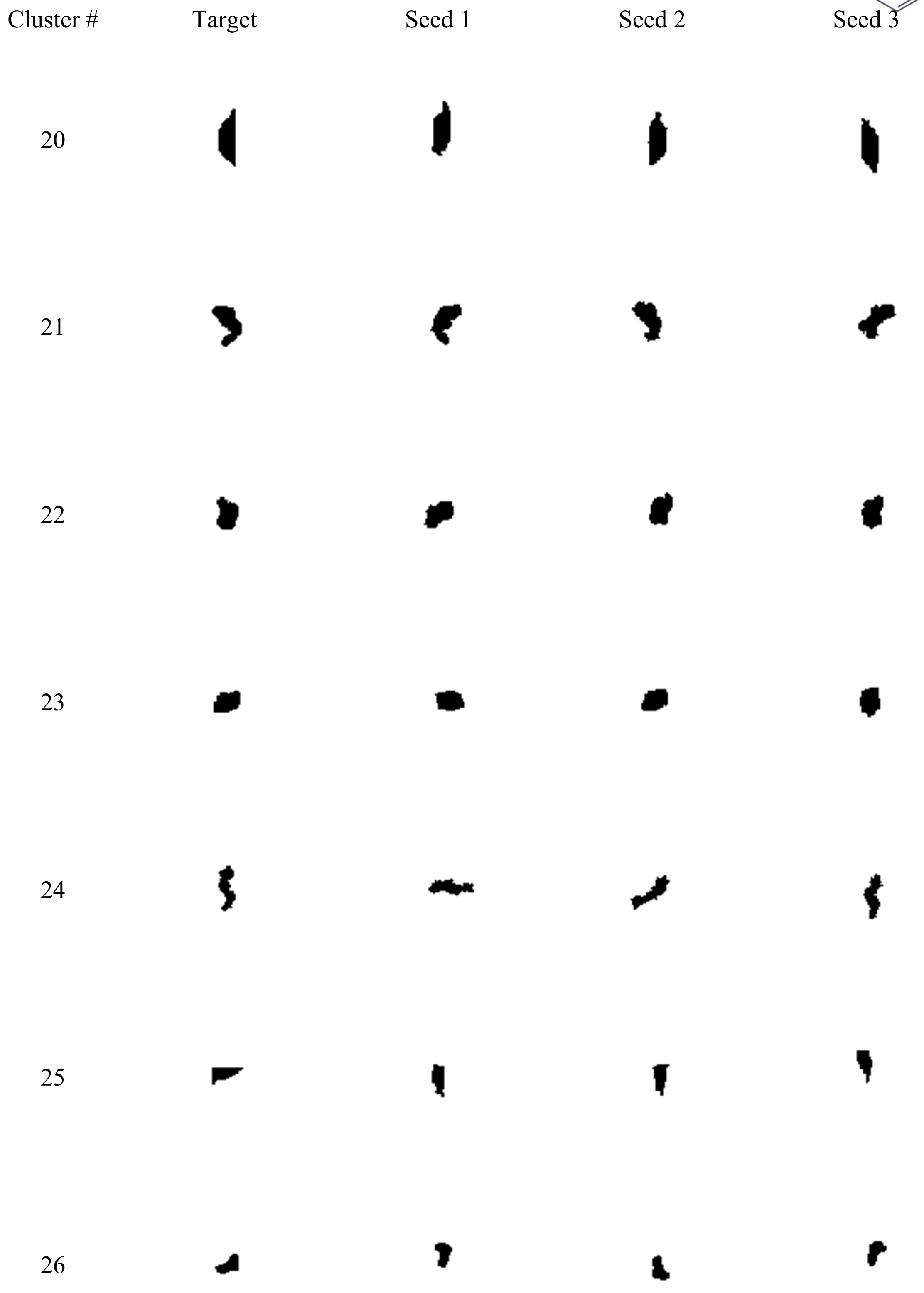

materials

MDPI

Cluster #	Target	Seed 1	Seed 2	Seed 3
27	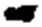	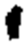	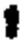	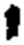
28	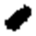	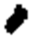	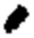	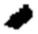
29	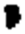	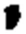	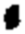	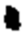
30	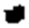	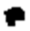	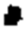	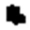
31	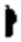	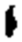	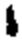	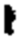
32	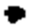	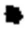	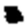	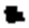
33	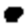	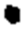	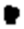	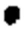

materials
MDPI

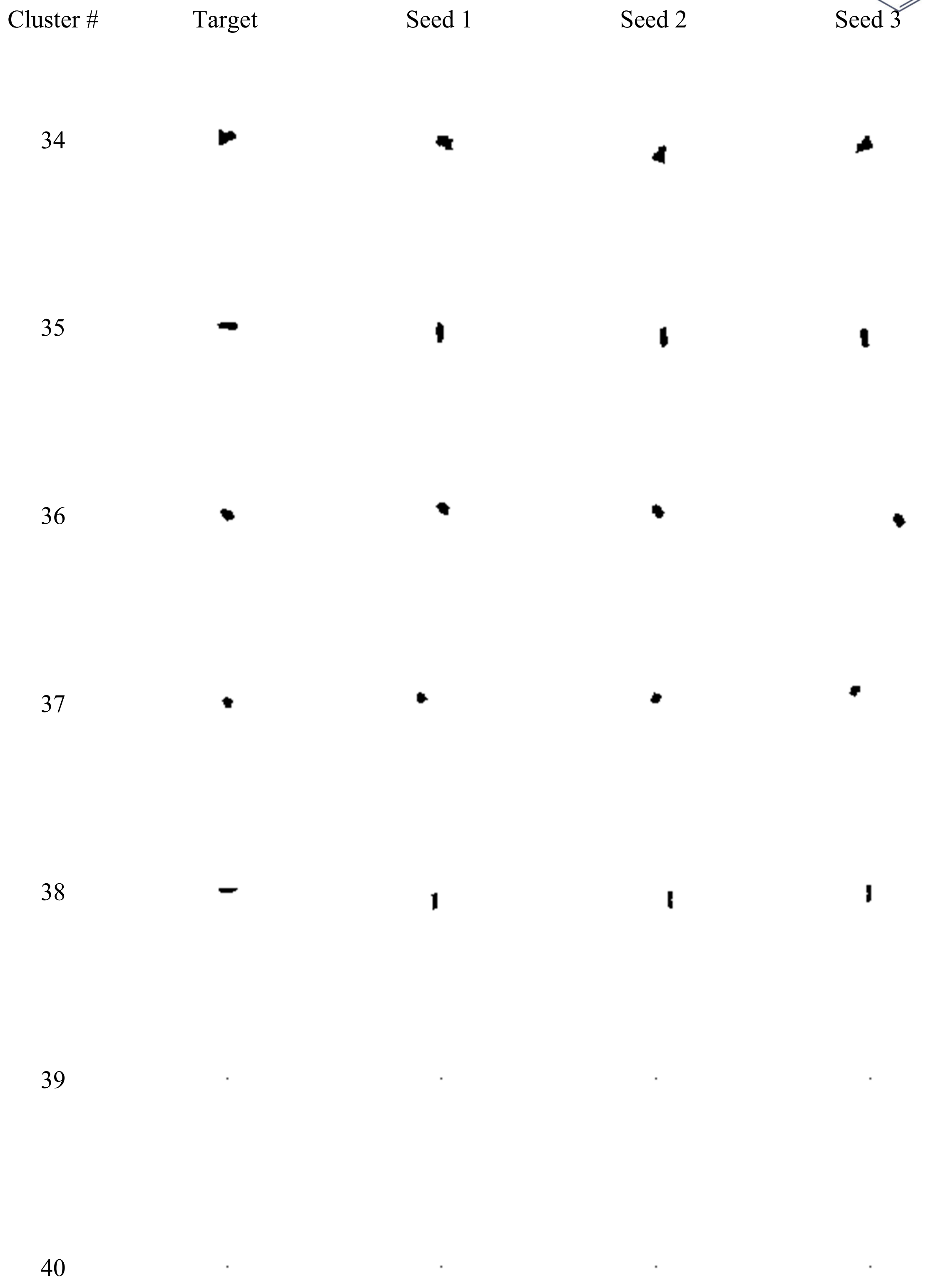

materials

MDPI

Cluster #	Target	Seed 1	Seed 2	Seed 3
41				
42				

© 2019 by the authors. Submitted for possible open access publication under the terms and conditions of the Creative Commons Attribution (CC BY) license (http://creativecommons.org/licenses/by/4.0/).
